# Association of Components of Severe Sarcopenia With Cognitive Decline in Older Adults: A Cross-Sectional Study

**DOI:** 10.7759/cureus.94121

**Published:** 2025-10-08

**Authors:** Nishant Roy, Debabrata Brahma, Anup Singh, Sanjay Gupta, Rameshwar N Chaurasia

**Affiliations:** 1 Department of Geriatric Medicine, Institute of Medical Sciences, Banaras Hindu University, Varanasi, IND; 2 Department of Psychiatry, Institute of Medical Sciences, Banaras Hindu University, Varanasi, IND; 3 Department of Neurology, Institute of Medical Sciences, Banaras Hindu University, Varanasi, IND

**Keywords:** appendicular skeletal muscle mass, correlates of cognitive impairment, gait speed, hand grip strength (hgs), six-meter walk test

## Abstract

Background

Among the components of sarcopenia, muscle strength and gait speed are strongly associated with cognitive performance, while the role of muscle mass remains unclear, particularly in severe sarcopenia. This study aimed to investigate the association of appendicular skeletal muscle mass (ASM), handgrip strength (HGS), and gait speed (six-meter walk test, 6MWT) with the Hindi version of the Mini-Mental State Examination (HMSE) in older adults with severe sarcopenia.

Methodology

In this hospital-based, cross-sectional study, 40 adults aged ≥60 years with HMSE scores ≤23 and severe sarcopenia (Asian Working Group for Sarcopenia criteria) were enrolled. Cognitive function was assessed using HMSE. ASM was measured by bioelectrical impedance analysis, HGS with a dynamometer, and gait speed using the 6MWT. The association between components of sarcopenia and HMSE scores was evaluated using Pearson’s correlation and multiple regression analyses.

Results

The mean age of the patients was 67.5 ± 3.39 years, with 70% being males (n = 28). Common comorbidities included hypertension (70%) and diabetes mellitus (50%). The mean HMSE score was 17.8 ± 1.98. ASM (r = 0.33, p = 0.032) and 6MWT (r = 0.51, p < 0.001) positively correlated with HMSE, whereas HGS showed a positive trend (r = 0.28, p = 0.07). In multivariate regression (adjusted), gait speed (β = 0.78, p < 0.001) and HGS (β = 0.40, p = 0.002) were strong predictors of HMSE, while ASM was the weakest predictor (β = 0.25, p = 0.034). Type 2 diabetes mellitus, hypothyroidism, and chronic kidney disease were significant negative predictors.

Conclusions

In severely sarcopenic older adults, gait speed and HGS are stronger predictors of cognitive decline than muscle mass. Simple assessments of gait and handgrip may help early detection of cognitive decline.

## Introduction

Sarcopenia and cognitive impairment are two of the major geriatric syndromes among older adults that can result in disability and loss of independence, with a prevalence of 43.6% and 7.4% respectively [1,2]. Cognitive impairment is characterized by difficulty in remembering, learning new things, concentrating, or making decisions that affect their everyday life [3]. A diagnosis of dementia is generally made when cognitive decline significantly impairs an individual’s ability to function socially or vocationally [3].

Sarcopenia, on the other hand, is characterized by a progressive age-related loss of skeletal muscle mass, strength, and physical function [1]. Several longitudinal and cross-sectional studies have reported that sarcopenia is associated with higher odds of mild cognitive impairment (MCI) and dementia [4-6]. For example, a three-year longitudinal study from China reported that individuals with sarcopenia had a significantly higher risk of developing MCI, with an odds ratio of 1.72 compared to those without sarcopenia [5]. Similar results that sarcopenia was associated with 1.74 times higher odds of MCI were found in an eight-year longitudinal study from Mexico [6]. Moreover, the individual components of sarcopenia, i.e., muscle mass, handgrip strength (HGS), and gait speed, are recognized not only as indicators of physical performance but also as potential markers of loss of cognition [7-12]. For example, a weaker HGS has been linked to poor memory, executive function, and processing speed [7,8]. While gait speed reflects the integrated function of motor, sensory, and cognitive systems, including frontal-subcortical circuits, cerebellum, and basal ganglia [9]. A slower gait speed is associated with an increased risk of cognitive impairment [10]. In contrast, the contribution of appendicular skeletal muscle mass (ASM) to cognitive outcomes remains unclear, with some studies showing gender-specific effects or weaker associations compared to other functional measures [11,12].

Although there is growing evidence of these associations, understanding these interrelationships is crucial for early identification and intervention in at-risk seniors [7-12]. Despite growing interest, few studies have focused specifically on severely sarcopenic older adults, a population at heightened risk for both physical and cognitive decline. According to the Asian Working Group for Sarcopenia (AWGS), severe sarcopenia is characterized by the presence of all three parameters of low muscle mass and low muscle strength and poor physical performance [13]. With this background, the present study aimed to investigate the association between ASM, HGS, six-meter walk test (6MWT), and cognitive impairment (Hindi version of the Mini-Mental State Examination (HMSE) scores ≤23) in severely sarcopenic older adults with cognitive decline in the Indian context.

## Materials and methods

Study design

This cross-sectional study was conducted at a tertiary care hospital in Northern India from January 01, 2024, to December 31, 2024. The study was approved by the Institutional Ethical Committee, Institute of Medical Sciences, Banaras Hindu University, Varanasi (approval number: Dean/2023/EC/4901; dated December 04, 2023).

Study participants

**Figure 1:**
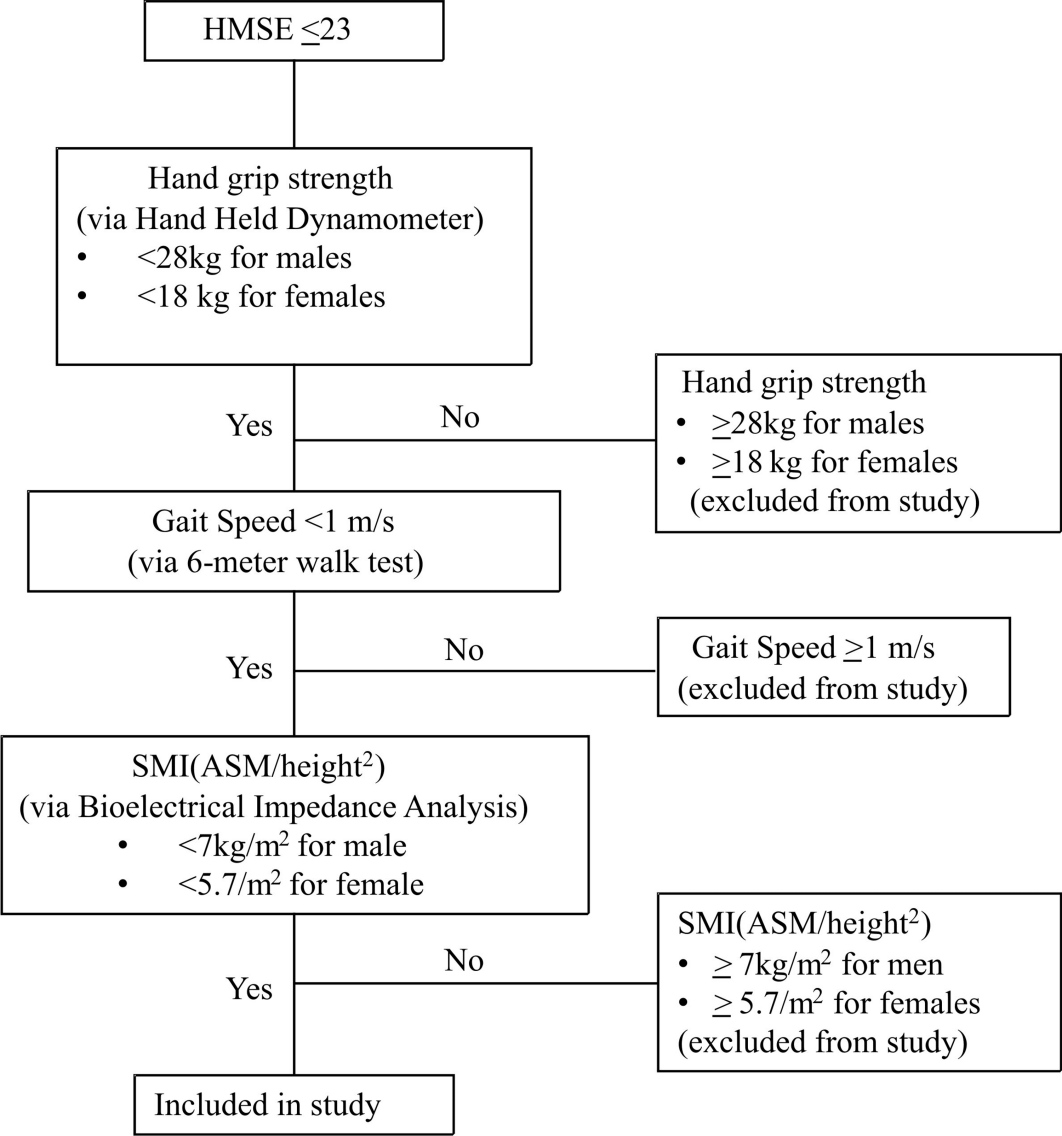
Algorithm used to screen and select study participants. HMSE: Hindi Mini-Mental State Examination; SMI: skeletal muscle index; ASM: appendicular skeletal muscle mass

A total of 40 individuals above 60 years of age of either gender with an HMSE score ≤23 were enrolled. The enrolled individuals were screened for severe sarcopenia according to the AWGS criteria [13]. The criteria are developed by the AWGS and are available as an open-access article under a Creative Commons Attribution-Non Commercial License (CC BY-NC 4.0), allowing for download, sharing, and non-commercial use with proper citation [13]. Individuals meeting the following criteria for severe sarcopenia were included in the study: HGS <28 kg for males or <18 kg for females, skeletal muscle mass index (SMI) <7.0 kg/m² for males or <5.7 kg/m² for females, and gait speed <1 m/second (Figure 1). Individuals satisfying all three above-mentioned criteria were categorized as severely sarcopenic. Individuals who were bedridden due to severe illness, had poorly controlled endocrine disease, had a history of psychiatric illness, and who denied informed consent were excluded.

Data collection

Demographic data of patients, including age, sex, and comorbidities, were obtained. Cognitive function was assessed using the HMSE. HMSE is a modified version of the Mini-Mental State Examination (MMSE) designed by Ganguli et al. for the illiterate population residing in India. HMSE is a freely available questionnaire with scores ≤23 indicating cognitive impairment [14]. ASM, HGS, and 6MWT speed were considered markers of physical performance. The ASM of the enrolled patients was measured using a validated bioelectric impedance analysis device. HGS was assessed using a handheld dynamometer, with the maximum value of three trials of the dominant hand, and the results were recorded in kilograms. The 6MWT was used to measure gait speed based on the time taken to walk six meters.

Outcome

The primary outcome was to understand the association between components of severe sarcopenia, i.e., HGS, ASM, and the 6MWT speed with cognitive impairment, as measured through the HMSE.

Statistical analysis

Data were analyzed using SPSS version 26.0 (IBM Corp., Armonk, NY, USA). Continuous variables were reported as mean ± standard deviation, and categorical variables as percentages. Normality was assessed using the Shapiro-Wilk test. Pearson’s correlation was used to evaluate relationships among ASM, HGS, 6MWT, and HMSE scores. Multiple regression adjusted for age, sex, and comorbidities (e.g., diabetes, hypertension) was analyzed. A p-value <0.05 was considered significant.

## Results

Demographic and clinical characteristics

Table 1 summarizes demographic and clinical characteristics of the 40 enrolled participants. The mean age was 67.5 ± 3.39 years (range = 61-74 years). The mean HMSE score was 17.8 ± 1.98, and 60% (n = 24) of the participants had moderate cognitive impairment (HMSE score = 10-19). In terms of physical parameters, the mean ASM was 11.99 ± 2.04 kg, and the mean HGS was 13.4 ± 2.85 kg. The 6MWT showed a mean speed of 0.48 ± 0.062 m/second.

**Table 1:** Demographic, clinical, and cognitive characteristics of the study participants (N = 40). ASM: appendicular skeletal muscle mass; HGS: handgrip strength; 6MWT: six-meter walk time; HMSE: Hindi Mini-Mental State Examination; SD: standard deviation; CKD: chronic kidney disease; CVA: cerebrovascular accident; RA: rheumatoid arthritis; T2DM: type 2 diabetes mellitus

Parameter	Category	n (%)/Mean ± SD
Age group (years)	60–65	16 (40%)
	65–70	20 (50%)
	>70	4 (10%)
Mean age (years) ± SD	-	67.5 ± 3.39
Sex	Male	28 (70%)
	Female	12 (30%)
Comorbidities	Hypertension	28 (70%)
	T2DM	20 (50%)
	Hypothyroidism	11 (27.5%)
	CVA	7 (17.5%)
	RA	1 (2.5%)
	CKD	1 (2.5%)
HMSE score	Mean ± SD	17.8 ± 1.98
HMSE score range	20–23 (mild impairment)	15 (37.5)
	10–19 (moderate impairment)	24 (60%)
	0–9 (severe impairment)	1 (2.5%)
ASM (kg)	Mean ± SD	11.99 ± 2.04
SMI (kg/m^2^)	Mean ± SD	4.94 ± 0.88
HGS (kg)	Mean ± SD	13.4 ± 2.85
6MWT (m/second)	Mean ± SD	0.48 ± 0.062

Relationship between HMSE scores and ASM, HGS, and 6MWT

An univariate Pearson correlation was performed between physical performance markers and HMSE scores, as shown in Table 2. ASM showed a moderate and statistically significant positive correlation with HMSE score (r = 0.33, p = 0.032). HGS was positively correlated with HMSE, although the association did not reach statistical significance (r = 0.28, p = 0.07). Among the functional measures, the 6MWT exhibited the strongest positive correlation with HMSE score (r = 0.51, p < 0.001).

**Table 2:** Pearson’s correlation of ASM, HGS, and 6MWT with HMSE scores. P-value <0.05 is considered statistically significant. ASM: appendicular skeletal muscle mass; HGS: handgrip strength; 6MWT: six-meter walk test; HMSE: Hindi Mini-Mental State Examination

Variables	r-value	P-value
ASM (kg)	0.33	0.032
HGS (kg)	0.28	0.07
6MWT (m/second)	0.51	<0.001

In the unadjusted regression analysis (Table 3), all three physical performance markers, i.e., 6MWT (β = 0.61, p < 0.001), HGS (β = 0.46, p < 0.001), and ASM (β = 0.31, p = 0.002), were found to be significant predictors of HMSE, with their predictive strength decreasing in that order.

**Table 3:** Focused linear regression analysis of physical performance markers on HMSE scores (unadjusted model). Model statistics: R² = 0.69; adjusted R² = 0.665; standard error = 1.14; observations = 40: F-statistics = 26.8; model p-value < 0.001. P-values <0.05 are considered statistically significant. ASM: appendicular skeletal muscle mass; HGS: handgrip strength; 6MWT: six-meter walk test; HMSE: Hindi Mini-Mental State Examination; SD: standard deviation; CI: confidence interval

Predictor	Unstandardized B	Unstandardized SE	Standardized beta (β)	t-value	P-value	95% CI (lower-upper)
ASM (kg)	0.302	0.090	0.31	3.34	0.002	0.118–0.484
HGS (kg)	0.323	0.064	0.46	5.06	<0.001	0.193–0.452
6MWT (m/second)	20.542	3.088	0.61	6.65	<0.001	14.280–26.803

In a multivariate regression model (adjusted), which included demographics (age and gender), comorbidities, and physical performance markers, i.e., 6MWT (β = 0.78, p < 0.001) and HGS (β = 0.40, p = 0.002), were found to be strong predictors of HMSE (Table 4). Contrastingly, ASM, while borderline significant (β = 0.25, p = 0.034), was the weakest predictor among the three. In the demographic and comorbidity profile, type 2 diabetes mellitus (β = -0.35, p = 0.013), hypothyroidism (β = -0.34, p = 0.010), and chronic kidney disease (CKD) (β = -0.24, p = 0.022) were found to be significant predictors of HMSE. In contrast, age, gender, hypertension, cerebrovascular accident, and rheumatoid arthritis did not show a statistically significant relationship with the HMSE score.

**Table 4:** Multiple linear regression of demographic, comorbidities, and physical performance markers on HMSE (adjusted model). Model statistics: R² = 0.806; adjusted R² = 0.73; standard error = 1.02; observations = 40: F-statistics = 10.62; model p-value < 0.001. P-values <0.05 are considered statistically significant. Gender (binary coding): male = 1; female = 0. Comorbidities (binary coding): 1 = present; 0 = absent. Includes hypertension, T2DM, hypothyroidism, CVA, RA, and CKD. ASM: appendicular skeletal muscle mass; HGS: handgrip strength; 6MWT: six-meter walk time; HMSE: Hindi Mini-Mental State Examination; SD: standard deviation; CI: confidence interval; RA: rheumatoid arthritis; CKD: chronic kidney disease; CVA: cerebrovascular accident; SE: standard error; T2DM: type 2 diabetes mellitus

Predictor	Unstandardized B	Unstandardized SE	Standardized beta (β)	t-value	P-value	95% CI (lower-upper)
Age	-0.088	0.067	-0.18	-1.30	0.204	-0.225–0.050
Gender	-0.222	0.553	0.05	-0.40	0.692	-0.913–1.356
Hypertension	-0.144	0.455	-0.03	-0.32	0.754	-1.078–0.788
T2DM	-1.429	0.545	-0.35	-2.62	0.013	-2.546–-0.314
Hypothyroidism	-1.471	0.535	-0.34	-2.75	0.010	-2.566–-0.376
CVA	-0.904	0.569	-0.17	-1.59	0.123	-2.11–0.23
RA	0.240	0.634	0.16	0.38	0.708	-2.74–6.93
CKD	-3.068	1.263	-0.24	-2.43	0.022	-5.65–-0.48
ASM (kg)	0.263	0.117	0.25	2.24	0.034	0.02–0.50
HGS (kg)	0.280	0.084	0.40	3.35	0.002	0.107–0.452
6MWT (m/second)	26.170	5.818	0.78	4.50	<0.001	14.25–38.09

## Discussion

Sarcopenia, apart from compromising physical function, also affects cognition in older adults. In a longitudinal study, conducted over eight years using the WHO Study on Global Ageing and Adult Health in Mexico, involving 496 adults aged ≥50 years, found that sarcopenia was associated with 1.74 times higher odds of MCI [6]. Similar results that sarcopenia is significantly associated with a higher risk of MCI (odds ratio = 1.72) compared to non-sarcopenia were found in another three-year longitudinal study from China [5]. A systematic review and meta-analysis of cross-sectional studies found that patients with sarcopenia had twice the odds of cognitive impairment (adjusted odds ratio = 2.2) [4]. In contrast to these associations, a study by Levine et al. found that sarcopenia was not associated with cognitive decline in individuals aged 60-69 years, whereas in those aged above 70 years, sarcopenia and obesity were independently associated with cognitive decline [15]. These contradictory results may be due to the components of sarcopenia assessed, i.e., muscle mass, muscle strength, and gait speed, which might play different roles in linking mental health and muscular health. This hospital-based study aimed to determine these associations in severely sarcopenic patients and understand how individual components of sarcopenia influence cognitive performance in older adults.

In our study, 70% of the participants with cognitive decline were males. Various studies have demonstrated that there is a higher prevalence of cognitive decline in females compared to males [16,17]. In addition to this, an eight-year longitudinal study found that women progressed at a faster rate from minimal cognitive impairment to Alzheimer’s disease compared to men [18]. While Peterson et al., in their study among 1,969 individuals, found that the prevalence of minimal cognitive impairment was higher among males compared to females [19]. The male predominance in our study was possibly due to the hospital-based design and underlying societal factors, where males are more likely to seek medical attention for functional and cognitive decline, whereas females may have limited access to healthcare or may underreport symptoms.

In our study, hypertension (70%) was the most prevalent comorbidity, followed by diabetes mellitus (50%). Similar observations have been reported in previous studies. For example, a case-control study by Wu et al., which enrolled 40,103 individuals aged >60 years, found that older adults with hypertension and diabetes (odds ratio = 1.53) have a higher risk of dementia compared to hypertension and diabetes alone [20]. Similarly, a longitudinal study by Ryuno et al. found that diabetes and a combination of diabetes and hypertension were risk factors for cognitive impairment [21]. Similarly, Wang et al. found that hypertension, followed by heart disease, dyslipidemia, diabetes, and cerebrovascular disease, were associated with patients with cognitive decline [22]. In our study, although hypertension and diabetes mellitus were most prevalent, in the regression analysis, type 2 diabetes mellitus (β = -0.35, p = 0.013), hypothyroidism (β = -0.34, p = 0.010), and CKD (β = -0.24, p = 0.022) were identified as significant negative predictors of HMSE scores, indicating their association with poorer cognitive performance.

Among the components of sarcopenia, muscle strength and gait speed appear to have the strongest and most consistent associations with cognition, particularly in domains such as executive function, memory, and processing speed. For example, a study by Gurholt et al., which involved 33,709 adults (aged 44-82), found that low muscle strength is significantly associated with reduced cognitive performance and brain structural changes, including lower white matter integrity, cortical thinning, and reduced brain volumes [23]. According to the National Health and Nutrition Examination Survey (NHANES) in the United States, Yang et al. stated that higher grip strength was significantly associated with memory, language, and attention [24]. Similarly, Sui et al., in their study among 329 men (aged 60-96 years), found that poor muscle quality (HGS/lean mass ratio) was associated with worse working memory, psychomotor speed, and visual learning [25]. Similar results that poor HGS was associated with poor cognitive outcomes were seen by Bai et al. [26]. The findings of our study aligned with the above findings. In the correlation analysis, patients with more severe cognitive impairment had weaker grips, although not statistically significant. However, in the regression analysis, HGS emerged as an independent predictor of cognitive function. Our finding that HGS is a strong predictor of cognitive decline suggests that it can be a simple, practical tool to assess cognitive risk in older adults.

Gait is one of the complex functions performed by the brain. It is not just a mechanical task; it requires the integration of motor, sensory, and cognitive systems (frontal-subcortical circuits, cerebellum, basal ganglia) [9,10]. In the current understanding, gait reflects not only musculoskeletal health but also cognitive control. For example, a longitudinal study involving 3,932 participants aged >60 years found that faster baseline walking speeds were at a lower risk of developing dementia [27]. Similarly, a systematic review and meta-analysis by Demnitz et al., which included 26 studies and 26,355 participants, found that a better gait speed is linked to superior cognition, executive function, memory, and processing speed [28]. Baseline slow gait, reflecting cognitive decline and predicted falls, was observed by Dyer et al. [29]. In our study, we found that gait speed measured by the 6MWT showed a significant correlation with HMSE (r = 0.47, p = 0.002). In the adjusted regression model, it was found to be a significant predictor (β = 0.78, p = <0.001) of cognitive performance. Clinically, incorporating brief gait assessments such as the 6MWT into routine geriatric evaluations may enhance early detection of cognitive decline and enable timely interventions.

The influence of muscle mass on cognitive decline remains unclear. For example, a longitudinal study for 5.6 years by Beeri et al., which involved 1,175 non-demented older adults at baseline, reported that muscle mass was not associated, but sarcopenia at baseline was associated with the incidence of dementia [30]. Another cross-sectional study by Bai et al. involving 428 older adults aged >80 years reported that muscle strength, not mass, was linked with MCI [26]. In contrast, a longitudinal study by Andrews et al. involving 1,704 older adults stated that lower appendicular lean mass (ALM) predicted higher dementia risk in older men but not women [12]. Similarly, another four-year prospective cohort study involving 515 older adults found that a decrease in muscle mass was associated with faster cognitive decline in men but not in women [11]. In our study, ASM, although significant (β = 0.25, p = 0.034), was the weakest predictor, reinforcing that muscle mass per se may be less relevant than muscle function for maintaining cognitive performance. Further exploration into the implications of ALM/ASM among the cognitively impaired population needs to be conducted.

The major strength of our study is that it is among the few Indian studies to examine both the geriatric syndromes, i.e., sarcopenia and cognitive impairment, in the same cohort. Further, few studies have specifically focused on severe sarcopenia in relation to cognitive impairment, particularly in the Indian population. This adds to the limited existing evidence on the association of individual components of severe sarcopenia with cognitive impairment.

Limitations

The major Limitations of our study include a relatively small sample size that restricts the generalizability of our findings, indicating a need for larger-scale studies to confirm these observations. Moreover, being a single-center study conducted at a tertiary care hospital, the results may not be representative of the diverse Indian population or elderly individuals residing in community settings. Finally, the cross-sectional design of this study precludes the establishment of causal relationships between the assessed variables. To explore the cause-and-effect dynamics between components of sarcopenia and cognitive function in older adults, longitudinal follow-up studies are strongly recommended.

## Conclusions

Slower gait speed and weak HGS were independently associated with lower HMSE scores. ASM also showed a positive association with cognition; however, its predictive strength was comparatively weaker compared to other parameters. The results support the use of simple, performance-based measures such as gait speed and HGS as practical, non-invasive screening tools in day-to-day geriatric and sarcopenia assessments to identify older adults at risk for cognitive decline. Future longitudinal studies with larger and more diverse samples are required to confirm these associations and explore the causal pathways linking sarcopenia components and cognitive decline in the Indian elderly population.

## Figures and Tables

**Figure 2:**
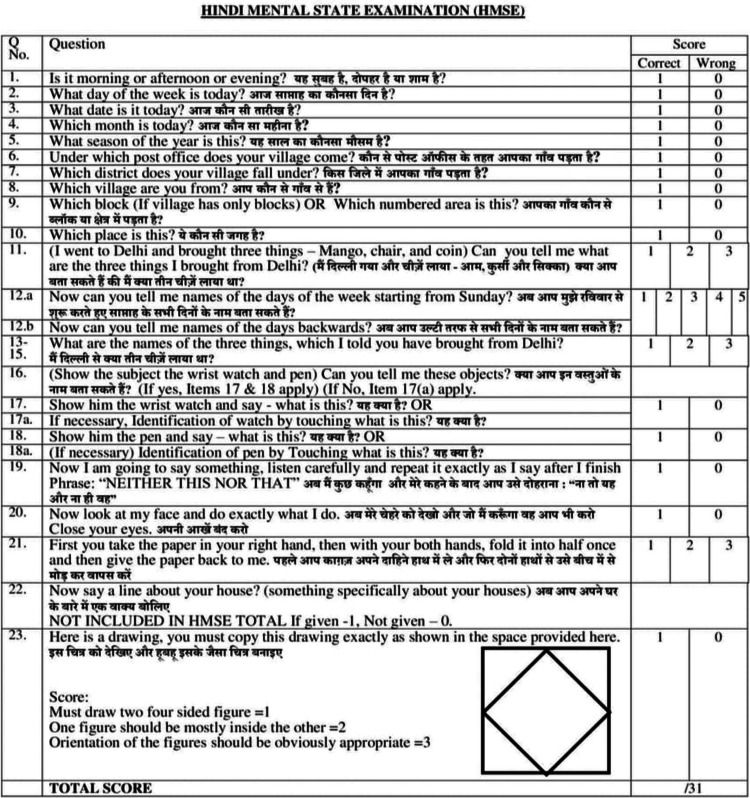
Hindi Mini-Mental State Examination (HMSE) questionaire for the assessment of cognition. HMSE scores ≤23 indicate impairment.
